# Deep-STP: a deep learning-based approach to predict snake toxin proteins by using word embeddings

**DOI:** 10.3389/fmed.2023.1291352

**Published:** 2024-01-17

**Authors:** Hasan Zulfiqar, Zhiling Guo, Ramala Masood Ahmad, Zahoor Ahmed, Peiling Cai, Xiang Chen, Yang Zhang, Hao Lin, Zheng Shi

**Affiliations:** ^1^Yangtze Delta Region Institute (Huzhou), University of Electronic Science and Technology of China, Huzhou, Zhejiang, China; ^2^Beidahuang Industry Group General Hospital, Harbin, China; ^3^Department of Plant Breeding and Genetics, University of Agriculture Faisalabad, Faisalabad, Pakistan; ^4^School of Basic Medical Sciences, Chengdu University, Chengdu, China; ^5^Innovative Institute of Chinese Medicine and Pharmacy, Academy for Interdiscipline, Chengdu University of Traditional Chinese Medicine, Chengdu, China; ^6^Clinical Genetics Laboratory, Clinical Medical College & Affiliated Hospital, Chengdu University, Chengdu, China

**Keywords:** snake toxin, deep learning, feature vectors, word embedding, feature selection, ANOVA

## Abstract

Snake venom contains many toxic proteins that can destroy the circulatory system or nervous system of prey. Studies have found that these snake venom proteins have the potential to treat cardiovascular and nervous system diseases. Therefore, the study of snake venom protein is conducive to the development of related drugs. The research technologies based on traditional biochemistry can accurately identify these proteins, but the experimental cost is high and the time is long. Artificial intelligence technology provides a new means and strategy for large-scale screening of snake venom proteins from the perspective of computing. In this paper, we developed a sequence-based computational method to recognize snake toxin proteins. Specially, we utilized three different feature descriptors, namely *g-gap*, natural vector and word 2 vector, to encode snake toxin protein sequences. The analysis of variance (ANOVA), gradient-boost decision tree algorithm (GBDT) combined with incremental feature selection (IFS) were used to optimize the features, and then the optimized features were input into the deep learning model for model training. The results show that our model can achieve a prediction performance with an accuracy of 82.00% in 10-fold cross-validation. The model is further verified on independent data, and the accuracy rate reaches to 81.14%, which demonstrated that our model has excellent prediction performance and robustness.

## 1 Introduction

**Figure 1:**
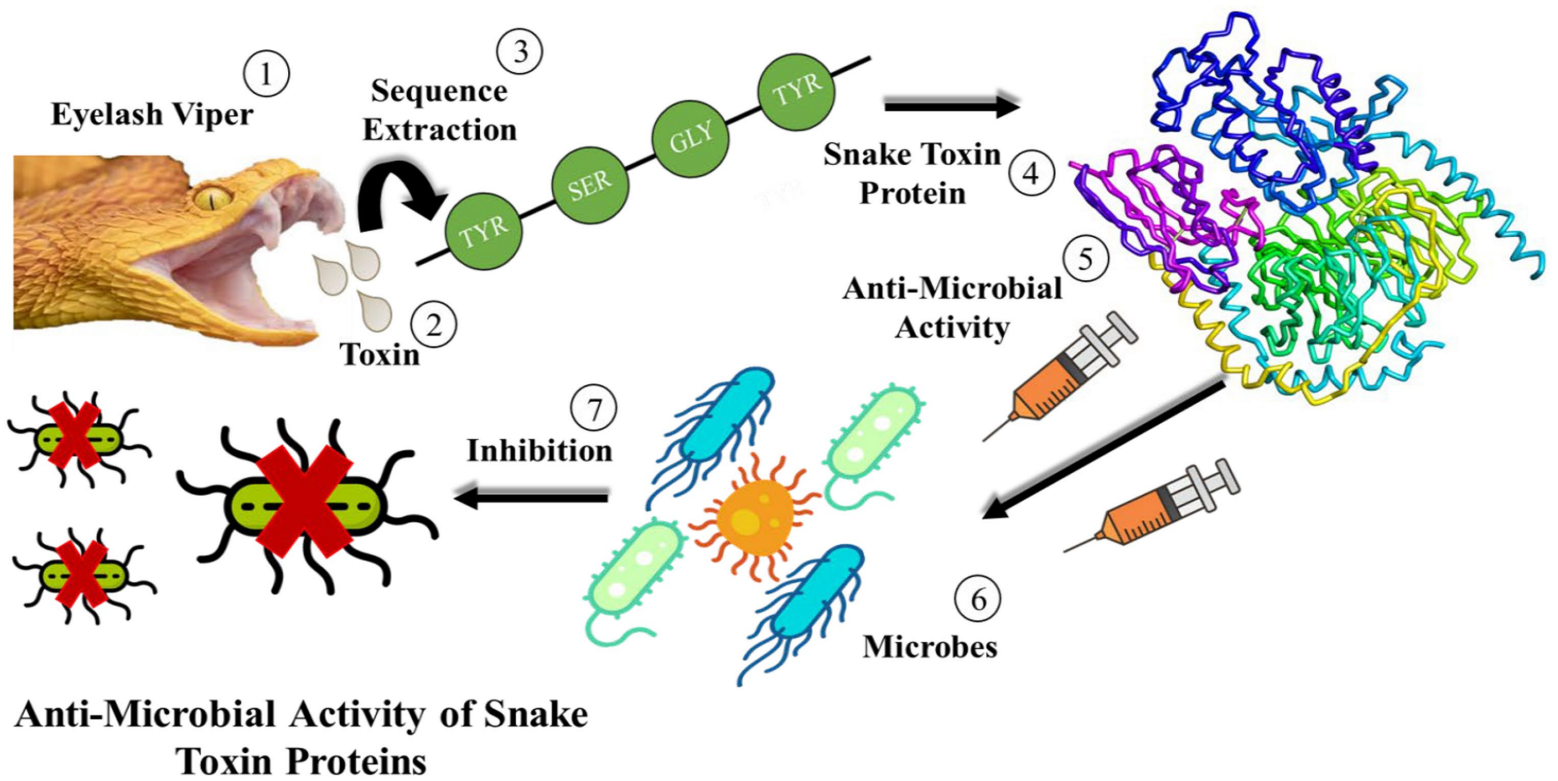
Schematic diagram of the anti-microbial activities of snake toxin proteins.

Snake venom is a mixture of toxin proteins and other chemical molecules, which acts on the blood circulation system, nervous system or motion system of prey. It can make the prey lose resistance, and then achieve the purpose of predation. Many toxin enzymes have been isolated from snake venoms, such as serine proteinases, metalloproteinase and L-amino acid oxidases, which can interrupt the blood circulatory system, leading to blood clotting and heart failure. Moreover, the scientists found that the primary toxins of *Pseudechis australis* venom with antibacterial activity were phospholipases A2 and L-amino acid oxidases. The L-amino acid oxidase discovered in the venom of *Crotalus adamanteus* was the first pure toxin tested against bacteria. Since then, crude snake venom, portions of it, or refined components have all shown antibacterial activity. The mechanism of anti-microbial activity of snake toxin proteins is shown in Figure 1.

Many toxin proteins were found in snake venom, such as phospholipases A_2_, cysteine-rich secretory proteins (CRISP), α-dendrotoxins, β-dendrotoxins and γ-dendrotoxins which could interact with nervous system or molecules in nervous system (1, 2). Scientists have also obtained some venomous proteins, for example, three finger α-neurotoxins (α-3FNTx) and acetylcholine esterase proteins, which target motion system of prey and cause paralysis (3). Surprisingly, the components extracted from snakes can be used as drugs to cure various diseases (4). At present, scientists have extracted several drugs from snake toxin proteins for the treatment of heart related syndromes. For example, captopril is now used to treat hypertension and reduce the risk of heart failure after the heart attack (5). Therefore, the correct identification of snake venom protein is very important for the study of drug development based on snake venom. Biochemical technologies are complicated, tedious and expensive. Thus, there is an urgent need to develop bioinformatic tools that can precisely identify snake toxins in a short time. Current bioinformatic tools, such as FASTA (6), HAlign (7, 8) and BLAST (9) can search for similar sequences with the help of known protein databases. However, in the absence of homologous sequences in benchmark dataset, these computational tools cannot correctly recognize snake toxin proteins. Therefore, it is essential to establish a computational tool to recognize snake toxin proteins.

**Figure 2:**
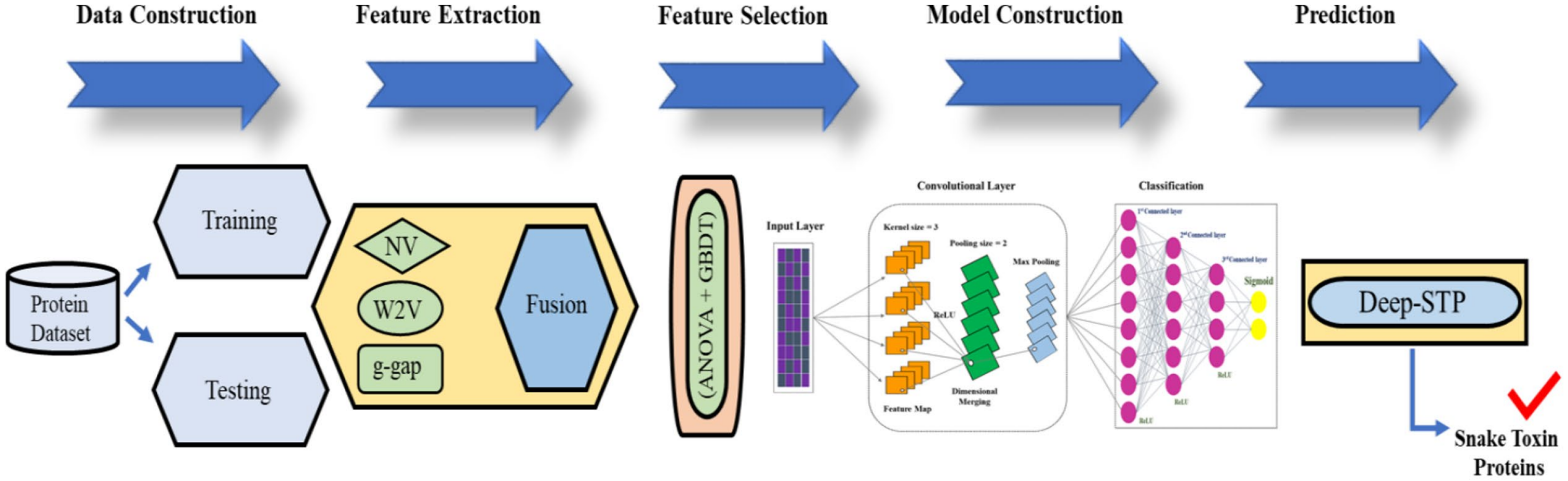
The graphical illustration of the entire study.

To fill the gap, we proposed the first predictor named Deep-STP based on deep learning to recognize snake toxin proteins. The graphical illustration of the entire study was shown in Figure 2. First, the snake toxin protein sequences were encoded by three different kinds of descriptors, namely, word to vector (10), *g-gap* and natural vector (11). Subsequently, the feature set was optimized by combining ANOVA (11) and GBDT (12) with IFS procedure. By inputting the optimal feature into deep learning, the snake toxin proteins can be recognized. The performance of the anticipated model was evaluated by 10-fold CV and independent data.

## 2 Materials and methods

A real and reliable data is crucial for the establishment of prediction model. In this work, positive and negative samples were collected from open-source database UniProt (13) and RefSeq (14). We have excluded the similar sequences using 80% as cutoff of sequence identity (15). After the elimination process, we finally obtained the dataset of 270 positive and 339 negative sequences of the prominent protein families of snake toxin. Subsequently, the data were separated into 80% training data and 20% independent data to objectively estimate the efficiencies and performances of the models, as shown in Supplementary Table S1.

### 2.1 Feature descriptors

It is an important step for protein function prediction to express the sequence information with effective mathematical descriptors (16). Here, three kinds of feature descriptors were used to encode the snake toxin protein sequences.

#### 2.1.1 g-gap dipeptide composition

The relationship between the two end-to-end 2-D amino acid residues can be expressed using this feature encoding approach. Consequently, important links between two residues are found using *g-gap* dipeptide composition. Thus, a protein ‘*F*’ can be described as


(1) $$F={\left[X_{1}^{p},X_{2}^{p},X_{3}^{p},X_{i}^{p},X_{400}^{p}\right]}^{t}$$

where ‘*t*’ is the transposition vector and $X_{i}^{p}$ is the *i*-th occurrence of *g-gap* dipeptide which is define as


(2) $$X_{i}^{p}=\frac{n_{i}^{p}}{L-p-1}$$

where ‘*p*’ is the number of amino acid residues, $n_{i}^{p}$ is the *i*-th value number of *g-gap* and ‘*L*’ is the length of ‘*F*’ protein.

#### 2.1.2 Natural vector

As a starting point for phylogenetic and evolutionary study, the natural vector scheme (NV) was created by Deng et al. (17). Here, we have also used NV to formulate the samples. A 60-dimensional vector can be created using this approach to plot biological sequences. The NV scheme has a significant ability to classify proteins because it has no parameters (18).

Let us say a protein ‘*P*’ with a length of ‘*L*’ residues can be expressed as.


(3) $$P=Q_{1}Q2\ldots Q_{i}\ldots Q_{L}$$

where *Q*i (i = (1, 2, … L)) indicates the i-th amino acid of protein ‘*P*’. The NV is expressed as.

w*_k_* (.): (A, C, D, E…W, Y) → (0,1).where w*_k_* (*Q_i_*) = 1, if *Q_i_ = k.* otherwise, w*_k_* (*Q_i_*) = 0.

In protein ‘*P*’, *m_k_* is the number of *k-*th amino acid which can be computed as


(4) $$mk=\overset{L}{\underset{i=1}{\sum }}wk\;\left(\mathrm{Qi}\right)$$

Let *T*_(*k*)(*i*)_ is the gap between the first and *i*-th amino acid, *η_k_* is the mean of the amino acids *k* and *S_k_* is the overall distance which is shown in equation (5).


(5) $$\left\{ \begin{array}{l} T_{\left(k\right)\left(i\right)}=i\times w_{k}\left(Q_{i}\right)\\ S_{k}=\overset{mk}{\underset{i=1}{\sum }}T_{\left(k\right)\left(i\right)}\\ \eta_{k}=S_{k}/m_{k} \end{array}\right.$$

Let ‘ $F_{2}^{k}$ ’ is the 2nd order regularized moment, which is computed as


(6) $$F_{2}^{k}=\overset{mk}{\underset{i=1}{\sum }}\frac{{\left(T\left(k\right)\left(i\right)-\eta k\right)}^{2}}{mk\times L}$$

Thus, ‘*P*’ can be termed as


(7) $$P={\left[m_{A},\eta_{A},F_{2}^{A},\ldots ,m_{R},\eta_{R},F_{2}^{Ri},\ldots m_{Y},\eta_{Y},F_{2}^{Y}\right]}^{T}$$

where ‘*T*’ is the vector transposition.

#### 2.1.3 Word2Vector

The ‘word2vector’ (W2V) is a NLP (Natural language processing) technique which has the ability to utilize neural networks to produce illustrations of the distribution of words (19, 20). In this method, word embeddings are utilized to illustrate of words. Indeed, the vectors which have the ability to encode the words closer in the vector space are supposed to be an identical meaning. The ‘word2vector’ consists of two different kinds of models, namely, continuous bag of words (21) and the other one is continuous skip gram (22). The main idea of the continuous skip gram is to utilize the words to predict its adjoining words (23). The quantified intelligence of continuous bag of words uses context words from a nearby booth to predict words. The continuous bag of words model structure logically implies the advantage of consistently condensing the scattered information in the data. Thus, in this work, we employed the continuous bag of words to train the appropriate resemblance of protein sequences. The dimension of the word2vector embedding is 200.

### 2.2 Feature selection

**Figure 3:**
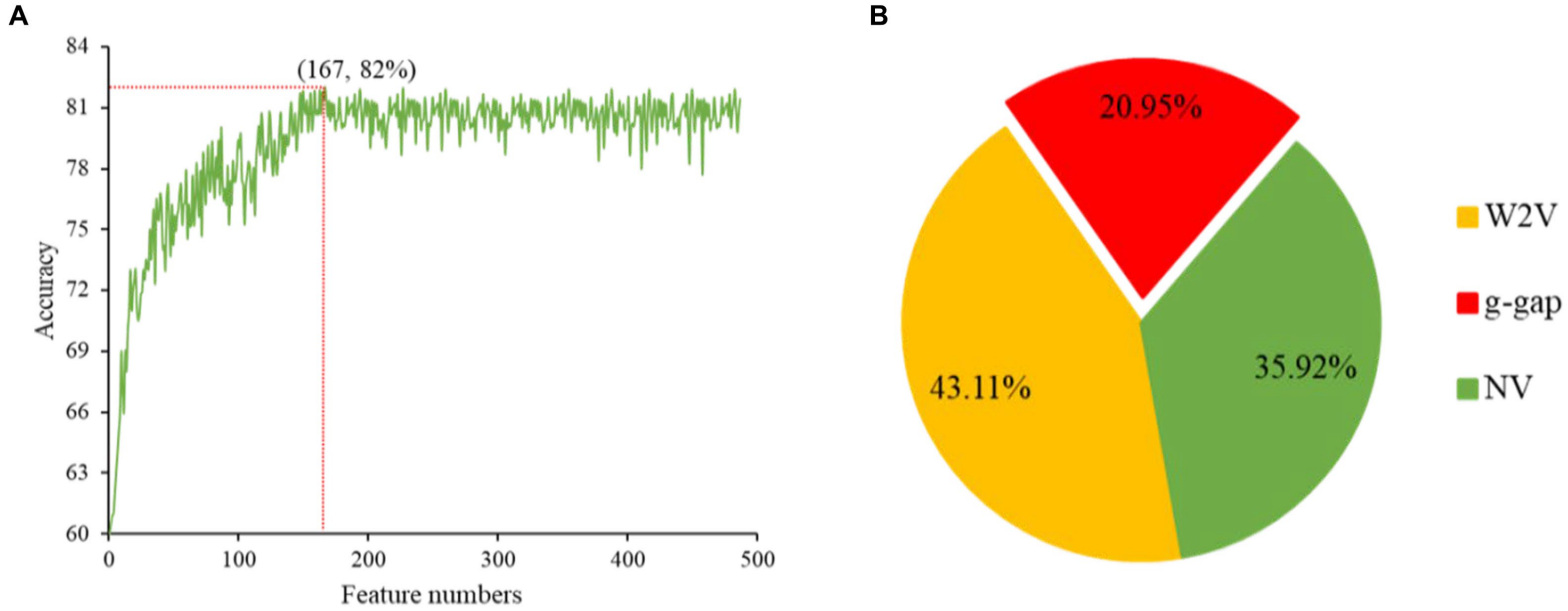
The prediction accuracy of models constructed with different numbers of features **(A)**. Contribution of descriptors in CNN-based fusion model to classify snake toxin proteins **(B)**.

The redundancy in the feature vectors can produce unsatisfactory performance (24). Therefore, selecting the ideal features is a significant step to eliminate the irrelevant features and enhance the efficiency of the model (25). There are many feature selection and ranking methods to optimize the features, such as ANOVA (26, 27), F-score (28), mRMR (29), GBDT and LGBM (12). ANOVA is a reputable choice to overcome these complications, because it takes short time and yield effective outcomes. The merging of top-performing features does not guarantee that the best outcomes can be achieved. These features are conceivably to have a higher level of redundancy, which leads to another unnecessary knowledge in the feature. Hence, GBDT is an ideal choice to conquer these hitches. In this work, ANOVA and GBDT with IFS were employed to achieve the best feature subset which could produce the maximum accuracy. The whole procedure for feature selection has been already elucidated in our previous study (12). The prediction accuracy of models constructed with different numbers of features and contribution of feature descriptors have been shown in Figures 3A,B.

### 2.3 Convolutional neural network

Convolutional neural networks (CNN) was first developed by LeCun et al. (30) and are now largely used in the developments of biology and bioinformatics (31). The core idea behind CNN is to use layer-wise convolutions and pooling techniques to build a large number of filters that can extract hidden topological properties from input. The performance of CNN on 2-D image and matrix data has been excellent (32). Moreover, 1-D CNN has been utilized to overcome the natural language processing and biomedical sequence data recognition problems (33). In this work, we executed 1-D CNN to recognize snake toxin proteins. We utilized Keras 2.3.1 (34), Python 3.5.4 and Tensor Flow 2.1.0 to execute this experimentation.

### 2.4 Metrics evaluation

Accuracy, precision, recall and F1-score (35) were used to assess the efficiency of the projected model and can be expressed as


(8) $$\left\{ \begin{array}{l} \mathrm{Precision}=\frac{TP}{TP+FP}\\ \mathrm{Recall}=\frac{TP}{TP+FN}\\ \mathrm{Accuracy}=\frac{TP+TN}{TP+FP+TN+FN}\\ F1=2\times \frac{\mathrm{Precision}\times \mathrm{Recall}}{\mathrm{Precision}+\mathrm{Recall}} \end{array}\right.$$

where ‘*TP*’ represents the truly predicted snake toxin protein sequences and ‘*FP*’ indicates the non-snake toxin protein sequences predicted as snake toxin protein sequence. ‘*TN*’ symbolizes the truly predicted non-snake toxin protein sequences and ‘*FN*’ demonstrate the snake toxin protein sequences which were predicted as non-snake toxin protein sequence.

## 3 Results and discussion

### 3.1 Performance evaluation

**Figure 4:**
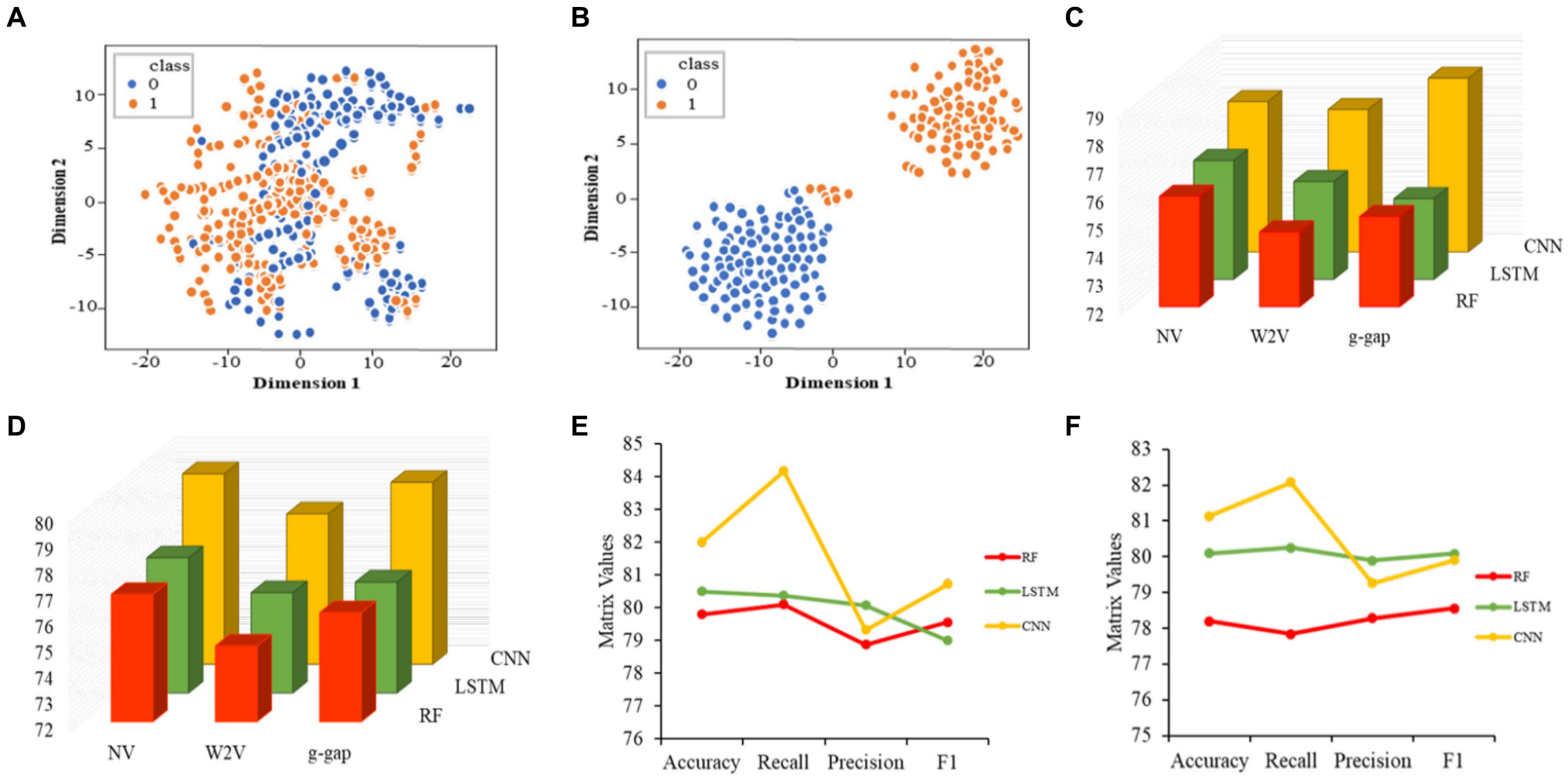
Visualization of feature fusion before the feature selection **(A)**. Visualization of feature fusion after the feature selection **(B)**. Performance of single-encoded features on different classifiers before the feature selection **(C)**. Performance of single-encoded features on different classifiers after the feature selection **(D)**. Comparison of proposed CNN-based fusion model with different machine learning-based fusion models on the basis of 10-fold CV **(E)**. Comparison of proposed CNN-based fusion model with different machine learning-based fusion models on independent data **(F)**.

Initially, we converted the sequence data into feature vectors by using three types of feature encoding schemes. Then, each feature vector was assessed by CNN-based classifier by employing a 10-fold CV. Subsequently, ANOVA and GBDT were implemented to select the optimal feature. Figure 3A displays the prediction accuracy of models constructed with different numbers of features. The maximum accuracy of 82.00% was achieved on 167 optimal features. Figure 3B shows the contribution of feature descriptors in CNN-based fusion model. The optimal model was trained on the data with 167 features derived from three kinds of descriptors. In final optimized-fusion model, NV, W2V and *g-gap* dipeptide descriptors account for 35.92, 43.11, and 20.95%, respectively. We have also visualized the feature fusions by using *t*-SNE (*t*-distributed stochastic neighbor embedding) technique. The *t*-SNE visualization of feature fusion before and after the feature selection are shown in Figures 4A,B. Figure 4C shows the single-encoding performance on different machine learning-based (ML-based) classifiers before the selection of features (36) and Figure 4D shows the performance of single-encoding after feature selections on different ML-based classifiers. Table 1 also shows the performance of feature fusion models before and after the feature selection on different ML-based classifiers by utilizing 10-fold CV.

**Table 1:** Performance of fusion models by using different algorithms.

Algorithm	FS	Dimension	Accuracy	Recall	Precision	F1	AUROC
RF	No	487	77.35	76.84	78.21	78.87	0.863
	Yes	189	79.80	80.10	78.88	79.56	0.881
LSTM	No	487	79.74	79.68	80.20	78.89	0.895
	Yes	227	80.50	80.37	80.08	79.00	0.901
CNN	No	487	81.22	83.11	78.01	79.88	0.904
	Yes	167	82.00	84.17	79.32	80.73	0.926

The comparisons of proposed CNN-based fusion model with different machine learning-based fusion models on 10-fold CV as well as on independent dataset are shown in Figures 4E,F. From these comparisons, we may conclude that the best model is based on the CNN with 167 optimal features. The model could produce the AUROC of 0.926 and 0.917 on training and independent dataset.

### 3.2 Performance evaluation of different ML algorithms

**Figure 5:**
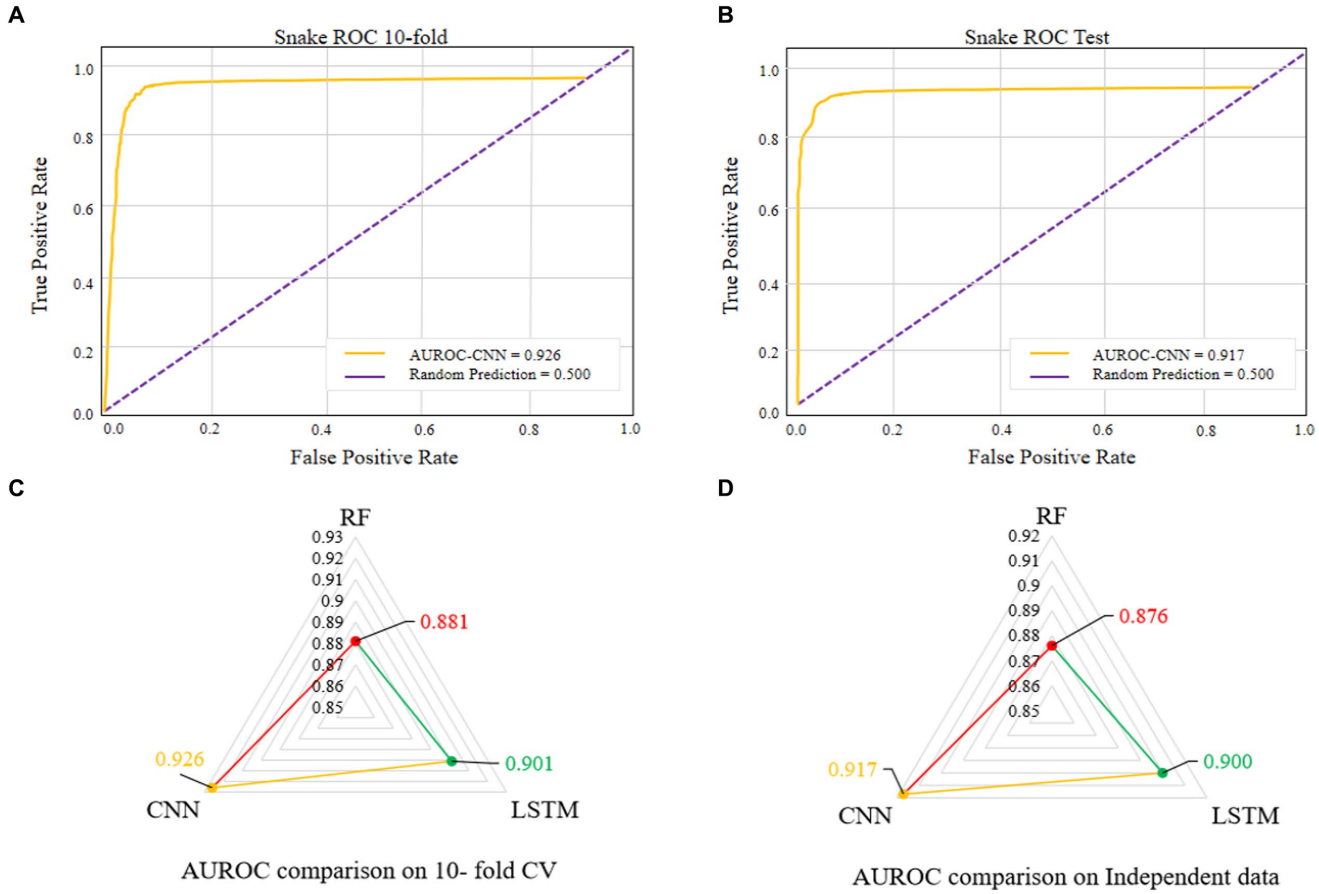
AUROC of the best performing model on 10-fold CV **(A)**. AUROC of the best performing model on independent data **(B)**. Comparison of different machine learning-based models on 10-fold CV **(C)**. Comparison of different machine learning-based models on independent data **(D)**.

Various single feature and their fusion were inputted into other ML-based classifiers, such as long short-term memory (LSTM) and random forest (RF), for determining which machine learning method is the best for snake toxin prediction. The 10-fold CV and independent dataset test were employed to estimate the efficiency of these models. The comparison outcomes have been shown in Tables 1, 2. We noticed that the AUROC of CNN-based prediction model was 2.5–4.5% higher than that of other classifiers on 10-fold CV and 1.7–4.1% higher than that of other classifiers on independent test. Figures 5A–D displayed that the CNN-based prediction model is best among all classifiers.

**Table 2:** Performance of fusion models on independent data.

Algorithm	Accuracy	Recall	Precision	F1	AUROC
RF	78.20	77.84	78.28	78.56	0.876
LSTM	80.10	80.25	79.89	80.09	0.900
CNN	81.14	82.08	79.26	79.91	0.917

## 4 Conclusion

Snake venom is a mixture of deadly proteins that can anesthetize and kill prey. Scientists have found a variety of proteins with potential pharmacological uses from snake venom. Further research on snake venom protein will contribute to drug development. In this work, an innovative computational model was constructed to classify snake toxin proteins. NV, W2V, and *g-gap* were utilized to encode the protein sequences. Subsequently, optimal feature subset was obtained by ANOVA and GBDT with IFS. By comparing different machine learning-based models, the best model was attained by the CNN-based classifier. Furthermore, the results showed that the proposed model could provide spectacular generalization ability. The dataset and codes are available at https://github.com/linDing-groups/Deep-STP. Further studies will focus on constructing a web application for the anticipated model. Moreover, other advance feature selection techniques and algorithms will be employed to further increase the efficiency of classification.

## Data availability statement

The original contributions presented in the study are included in the article/Supplementary material, further inquiries can be directed to the corresponding authors.

## Author contributions

HZ: Conceptualization, Experimentation, Methodology, Visualization, Writing—original draft preparation. ZG: Data curation, Methodology, Experimentation. RMA: Data curation, Experimentation, Methodology, Visualization. ZA: Data curation, Methodology, Visualization. PC: Data curation, Visualization. XC: Methodology. YZ: Methodology, Writing – review & editing. HL: Conceptualization, Supervision, Writing – review & editing. ZS: Conceptualization, Writing – review & editing. All authors have read and agreed to the published version of the manuscript.
