# Supplementary material for: Deep-STP: a deep learning-based approach to predict snake toxin proteins by using word embeddings
Source: Front Med (Lausanne). 2024 Jan 17;10:1291352. doi: 10.3389/fmed.2023.1291352 (PMC10829051; doi:10.3389/fmed.2023.1291352)
Supplement: Supplementary file 1 [file Table_1.DOCX]

**Table 1S.** The distribution of sample numbers in the dataset.

| Attribute | Protein Family | Training Data | % age | Independent Data | % age | Total |
| --- | --- | --- | --- | --- | --- | --- |
| Positive | Metalloproteinase | 216 | 10.64 | 54 | 46.29 | 270 |
|  | Serine Proteases |  | 25.46 |  | 9.250 |  |
|  | Phospholipases |  | 35.64 |  | -- |  |
|  | 3-Finger Toxin  L-amino-acid oxidase  Metalloproteases  Snaclec  C-type lectin  Natriuretic peptide  Snake venom vascular endothelial growth factor  Venom phosphodiesterase 1  Acetylcholinesterase  Venom factor  Muscarinic toxin 7  Cysteine-rich venom protein pseudechetoxin  C-type lectin  Lebetin-2-alpha  Venom nerve growth factor  Kunitz-type neurotoxin MitTx-alpha  Protease inhibitor 4  Waglerin-4  Cytotoxin 4  Bradykinin-potentiating peptide  Crotamine-IV-2 |  | --  1.388  0.462  6.944  1.851  8.796  2.314  0.462  0.462  0.462  0.462  0.462  1.851  0.462  0.925  0.462  0.462  --  --  --  -- |  | 12.96  --  1.851  --  --  --  --  --  --  --  --  --  --  --  --  --  --  1.851  1.851  24.07  1.851 |  |
| Negative | Metalloproteinase | 271 | 0.369 | 68 | -- | 339 |
|  | Serine Proteases |  | 4.059 |  | 19.11 |  |
|  | Phospholipases |  | 17.54 |  | 1.470 |  |
|  | 3-Finger Toxin  Azemiopsin  Snaclec  Poly-His-poly-Gly peptide 2  Sarafotoxin  Nigwaprin-a  Nawaprin  Waprin  Vascular endothelial growth factor A  Ohanin  5'-nucleotidase  Pro-rich toxin 4  Translationally-controlled tumor protein homolog  Short neurotoxin  Long neurotoxin  Cytotoxin  Cysteine-rich venom protein  Cystatin  Bitiscystatin  Cytochrome  Serotriflin  Alcohol dehydrogenase 1  Calglandulin  Actin, alpha skeletal muscle  Mambalgin-1  Bradykinin-potentiating peptide  Cathelicidin-related peptide  Neurotoxin  Weak toxin  Ringhalexin  Alpha-elapitoxin  Frontoxin  Denmotoxin  Micrurotoxin  Muscarinic  Beta-cardiotoxin  Adrenergic toxin  Clarkitoxin  Fulgimotoxin  Bucandin  Acetylcholinesterase toxin  Thrombostatin  Pseudonajatoxin b homolog  Alpha-colubritoxin  weak neurotoxin  Gamma-bungarotoxin  Cardiotoxin-like protein  Bucain  Haditoxin  Alcohol dehydrogenase  Cathelicidin-related peptide  Calglandulin  Disintegrin  Fused toxin protein  Ryncolin  Antihemorrhagic factor  HSF-like protein  Hyaluronidase  Insulin  1370.6 Da venom vasodilator peptides  1095.6 Da venom vasodilator peptide  Alpha-bungarotoxin  Kappa-bungarotoxin  Toxin Lc b  Vespryn  Cobrotoxin  Rho-elapitoxin  Synergistic-type venom protein  Toxin S4C8  Alpha-elapitoxin  Ringhalexin  Toxin C13S1C1  Toxin F-VIII  Toxin S5C10  Toxin C10S2C2 |  | 4.059  --  7.380  --  --  --  0.369  1.107  --  --  --  --  --  5.904  5.904  3.321  2.952  1.107  0.369  0.369  0.369  0.369  0.369  0.369  0.369  0.369  0.738  0.738  2.214  0.369  3.690  1.107  0.369  0.369  1.845  0.369  0.369  0.369  0.369  0.369  0.369  0.369  0.369  0.369  1.476  0.369  0.369  5.904  0.369  0.369  0.738  0.369  5.904  0.369  0.738  0.738  0.369  0.369  0.369  0.369  0.369  0.369  0.369  0.369  0.369  0.369  0.369  0.369  0.369  0.369  3.690  0.369  0.369  0.369  0.369  0.369 |  | --  1.470  58.82  1.470  2.941  1.470  --  4.411  2.941  1.470  1.470  1.470  1.470  --  --  --  --  --  --  --  --  --  --  --  --  --  --  --  --  --  --  --  --  --  --  --  --  --  --  --  --  --  --  --  --  --  --  --  --  --  --  --  --  --  --  --  --  --  --  --  --  --  --  --  --  --  --  --  --  --  --  --  --  --  -- |  |
| Total |  | 487 |  | 122 |  | 609 |
